# Does a PBL-based medical curriculum predispose training in specific career paths? A systematic review of the literature

**DOI:** 10.1186/s13104-016-2348-0

**Published:** 2017-01-07

**Authors:** Jordan Tsigarides, Laura R. Wingfield, Myutan Kulendran

**Affiliations:** 1 James Paget University Hospital, Lowestoft Road, Gorleston-on-Sea, Great Yarmouth, Norfolk, NR31 6LA UK; 2 University Hospital Southampton, Southampton, UK; 3 Department of Surgery, St. George’s Hospital, London, UK

**Keywords:** Problem-based learning, Career choice, PBL, Medical education, Residency, Primary care

## Abstract

**Background:**

North American medical schools have used problem-based learning (PBL) structured medical education for more than 60 years. However, it has only recently been introduced in other medical schools outside of North America. Since its inception, there has been the debate on whether the PBL learning process predisposes students to select certain career paths.

**Objectives:**

To review available evidence to determine the predisposition of specific career paths when undertaking a PBL-based medical curriculum. The career path trajectory was determined as measured by official Matching Programs, self-reported questionnaires and surveys, and formally defined career development milestones.

**Methods:**

A systematic literature review was performed. PubMed, Medline, Cochrane and ERIC databases were analysed in addition to reference lists for appropriate inclusion.

**Results:**

Eleven studies fitting the inclusion criteria were identified. The majority of studies showed that PBL did not predispose a student to a career in a specific speciality (n = 7 out of 11 studies, 64%). However, three studies reported a significantly increased number of PBL graduates working in primary care compared to those from a non-PBL curriculum.

**Conclusions:**

PBL has been shown not to predispose medical students to a career in General Practice or any other speciality. Furthermore, a greater number of similar studies are required before a definitive conclusion can be made in the future.

## Background

Medical education continues to grow and evolve as the demands of both doctors and patients change. Problem-based learning (PBL) structured medical education has been used by North American medical schools for more than 60 years but has only been introduced to other global medical schools within the last 20 years [1]. Now, PBL is used in medical schools all over the world and it ‘appears to have become the preferred pedagogical strategy in tertiary education worldwide’ [2]. Research into problem-based learning compared with traditional curricula has revealed no difference in knowledge levels between programmes. The evidence demonstrates that PBL may increase collaboration and self-directed learning [3]. The current research in PBL programme evaluation has generally focused on outcome measures such as examination scores, supervisor assessments, and student satisfaction [4]. However, there are a relatively small number of studies into how the PBL learning process predisposes students to select certain career paths.

The theory behind PBL lies in creating a curriculum that orientates students towards lifelong learning and a realistic approach to knowledge accruement. It was originally designed and piloted by two North American medical schools in the 1950s and 1960s, Case Western Reserve University and McMaster University. Proponents of the approach argue that it has been the most important innovation in professional education [5]. Driven by what was seen as a gap in the education being offered at McMasters to students during their neurology clinical rotations, Barrows and Tamblyn worked to create a PBL-based programme. They believed it allowed students an opportunity to integrate knowledge across subjects and simultaneously learn critical problem-solving skills. Shortly after the integration of PBL into medical curricula, other professions including nursing and engineering began to adopt PBL [6, 7].

An increasing demand for general physicians in the US, UK, and many other countries strains healthcare systems. Medical organisations are calling for reforms within medical education to encourage future doctors to enter a generalist training path [8]. Research has shown that certain educational variables including early exposure to family medicine correlate with graduates entering generalist careers. Matsui et al. [9] researched Tokyo medical students and showed a propensity within PBL graduates for an interest in primary care compared with those following a traditional curriculum. Research in the United States showed similar outcomes, with Peters et al. [10] demonstrating that Harvard medical students exposed to PBL-based learning were more likely to practice primary care or psychiatry compared with students not enrolled in the innovative curriculum. Although this study reviews the impact of PBL-based education on career selection, it is acknowledged that the final career choice for doctors is extremely multifactorial. A large Norwegian-based study by Wesnes et al. [11] highlighted key demographic data that influenced a general physician career choice including sex, age, the location of study, and citizenship. Whilst many variables that influence career choice are not modifiable, curriculum implementation is a relatively easy variable to alter. This could directly translate to what types of doctors are produced to meet increasing demands in certain specialities (hospital versus community-based physicians). Ultimately, students could be separated into different curriculums based on their career intentions.

To date, there has been no review with the single focus to determine if PBL-based medical education influences students to pursue a specific career path. The aim of this study was to perform a systematic review of the effect of PBL-based medicine versus traditional lecture-based courses in medical career choice.

## Methods

### Literature search strategy

We identified original studies on PBL-based curriculum and medical career choice by searching the following databases: MEDLINE, PubMed Central, Cochrane, and ERIC, databases on 21st July 2015. These publications were in the English language and we placed no date limitations. We used the following keywords and phrases for BOTH: problem-based learning or PBL, medical school AND problem-based learn*, PBL, tutor based learning AND medical student*, PBL AND medical educat*, career and medical graduates. Following this, we then further manually reviewed the reference lists from studies retrieved from the above search to identify other potentially relevant studies.

### Selection criteria

Papers focusing solely on academic performance, nationalised test results and student demographic were excluded from the review. Furthermore, we excluded studies that did not discuss PBL-based programmes. We made no exclusions on country of curriculum origin. The PBL group size was not limited, however, most groups included within this research ranged from 6–15 students. Two of the authors (JT and LW) independently identified relevant abstracts, the selection of studies based on the criteria described above, and the subsequent data abstraction, with the senior author (MK) resolving any conflicts.

### Critical analysis

Following selection, each of the studies were critically analysed. The widely used STROBE [12] and CONSORT [13] quality assessment checklists were used to aid with analysis of observational studies and randomised controlled trials respectively.

## Results

### Literature review

**Fig. 1:**
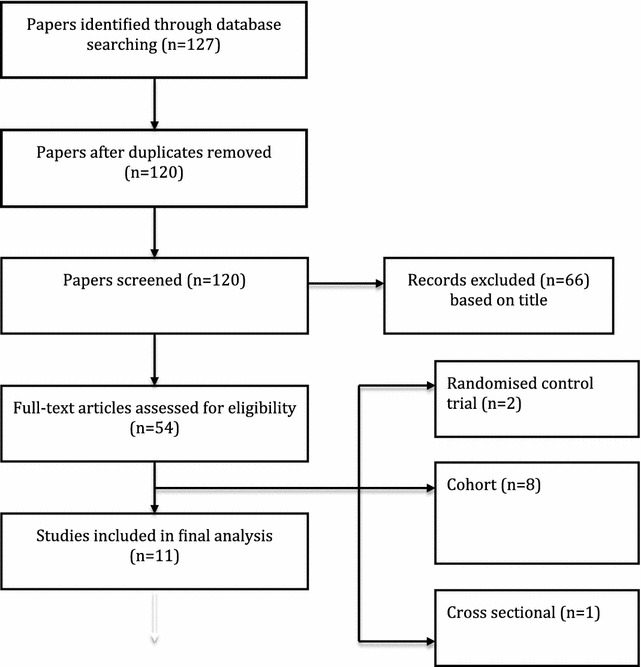
Flow chart illustrating the selection of papers included in the review and the papers excluded with a brief reason for exclusion

Out of 127 initial studies via the search terms, seven duplicate studies were removed. The remaining 120 article titles were screened for suitability within this study. A further 54 were selected for eligibility for full paper review. From this group of articles, 43 were excluded, as they did not focus on PBL specific curricula or focused on student demographics as predictors of medical specialities (Fig. 1). Of the 11 studies reviewed, eight studies were conducted in participant cohorts in North America (Canada and the USA), two in Australasia (Japan and Australia), and a further one in Europe (Norway).

### Participants

A summary of the characteristics of included studies is presented in Table 1. Wide inclusion criteria meant that studies reviewed were published over a span of 27 years (range = 1985–2012). The studies published prior to 2000 were all based in Canada or the USA, as medical schools in these countries were the first to adopt PBL as part of their curricula. The later studies showed PBL curricula in Japan, Australia, and Norway.

**Table 1:** Summary of study characteristics including demographics and outcome measures

Reference (year)	Country and school	Study design	Total number of participants	Programs compared (number in each group)	Level of training (graduation years)	Other relevant demographics/adjustment for confounding	Outcome measures
Ford et al. [4]	Canada. All English-language schools	Retrospective cohort	14,370	PBL vs non-PBL^a^ (6391/7979)	Graduates (1993–2004)	None	Pathology residency choice
Kaufman et al. [14]	USA. University of New Mexico School of Medicine	Retrospective cohort	511	Primary care (PBL) vs traditional curriculum (140/379)	Final year medical students	None	Future specialty choice
Matsui et al. [9]	Japan. Tokyo Women’s Medical University	Retrospective cohort	468	PBL vs Non-PBL curriculum (248/220)	Graduates (1989–2003)	All female, aged between 25–53	Specialty choice
Mennin et al. [17]	USA. University of New Mexico School of Medicine	Retrospective Cohort	120	Conventional vs primary care (PBL) curriculum (87/33)	Graduates (1983–1986)	None	Family practice specialty
Moore et al. [15]	USA. Harvard Medical School	Randomised controlled trial	297	New pathway (PBL) vs traditional curriculum (62/235)	Graduates (1989–1990)	No significant difference between groups in terms of age or gender	Residency choice
Moore-west et al. [16]	USA. University of New Mexico School of Medicine	Cohort study	38	New pathway (PBL) vs traditional curriculum (19/19)	Graduates (1983–1984)	None	Residency choice
Pearson et al. [18]	Australia. University of Newcastle and University of Sydney	Retrospective cohort	2469	PBL vs Non-PBL^b^ (538/1931)	Graduates (1983–1998)	Matched for graduation year, age and gender	Specialty choice
Peters et al. [10]	USA. Harvard Medical School	Randomised Controlled Trial	100	New Pathway (PBL) vs traditional curriculum (50/50)	Graduates (1989–1990)	Matched for age & gender	Specialty choice
Tolnai et al. [20]	Canada. McMaster University and University of Ottawa	Retrospective Cohort	342	PBL vs Non-PBL^b^ (156/186)	Graduates (1974–1980)	None	Family practice specialty
Wesnes et al. [11]	Norway. all Norwegian Medical Schools	Cross-sectional	1770	PBL vs Non-PBL^b^	Graduates (2002–2005)	None	General practice specialty
Woodward et al. [19]	Canada. McMaster University and other Canadian Universities	Retrospective cohort	2028	PBL vs Non-PBL^b^ (408/1620)	Graduates (1972–1979)	Group 1: matched on year of graduation. Group 2: matched on year of graduation, sex and age	Specialty choice

^a^PBL schools were defined as such following interviews with representatives from each of Canada’s 13 english-language medical schools

^b^Graduates from schools with a mainly PBL based curriculum were compared with those from schools with a traditional non-PBL based curriculum

The sample size of the studies ranged greatly. The mean number of participants was 2047 (38–14,370). There was a propensity towards surveying already practising physicians (n = 22,002), although Kaufman et al. [14] used final year medical students (n = 511). Matsui et al. [9] looked at a cohort of female medical school graduates who had graduated from Tokyo Women’s Medical University over a series of years (1989–2003). Twenty-seven percent of studies (3/11) highlighted within their research that age and gender were matched. Of these, two were retrospective cohort studies and one was a randomised controlled trial. None of the studies included additional demographic data on the ethnic background of participants or additional potential confounders such as previous degrees and family makeup (i.e. physicians with children, etc.).

### Outcome

All of the included studies directly compared outcomes between participants from a PBL based curriculum to those from a non-PBL based curriculum.

The outcome measures for all studies within this review were specifically looking at the medicine speciality selection of students/graduates. Three out of eleven studies’ end points were if PBL influenced a career choice in family medicine/general practice with one further study looking specifically at a Pathology residency choice.

### Measurement tools

Outcome measures were assessed using a survey/interview-based approach in ten out of eleven studies with only Wesnes et al. [11] solely taking data from a membership database (Norwegian Medical Association physician membership database). Ford et al. [4] used data from the Canadian Resident Matching Service (CaRMS) on programme rankings. The questionnaires focused on demographics, curriculum type studied and practice outcomes, among other variables that are not relevant to this review.

### Study design

Within the included papers, seven were a retrospective cohort, one was a prospective cohort, one was a cross-sectional and two were randomised control trials (RCT). The two RCTs (Moore et al. [15] and Peters et al. [10]) used the same cohort of graduates from Harvard Medical School between 1985 and 1987. During this time, the programme ran parallel tracts (PBL vs. Non-PBL) and participants were randomised to one of the tracts. Moore et al. [15] used multiple questionnaires to assess students’ career preference throughout medical school and a further exit questionnaire when students graduated to assess their residency choice. In comparison, Peters et al. [10] followed up this cohort 12 and 13 years post-graduation to assess their career choices in a longer term follow-up study. Moore-West et al. [16] conducted a prospective cohort study at the University of New Mexico, which compares student career preference and final residency choice from PBL vs. Non-PBL curricula. Following this Mennin et al. [17] conducted a retrospective cohort study on the same study population 4–7 years post graduation, gaining information on career choice.

Five studies gained data from a database. Ford et al. [4] gained data from the Canadian Resident Matching Service (CaRMS) over a 12 year period (1993–2004) to determine the number of graduates in each cohort ranking a Pathology programme first. Matsui et al. [9] used data gained from the Japanese Ministry of Health to form controls. Pearson et al. [18] used the New South Wales medical board to identify participants. Wesnes et al. [11] conducted a cross-sectional study and used the Norwegian Medical Association Database to gain data on graduates over a 4 year period (2002–2005). Finally, Woodward et al. [19] used the Canadian Medical Association Physician Manpower Databank to gain data on graduates from other Canadian English-language medical schools for comparison with McMaster graduates.

Five of the included studies gained data on graduates from multiple medical schools with different curricula. Ford et al. [4] used participants from 13 medical schools in Canada. Woodward et al. [19] also used participants from ‘other’ Canadian medical schools as a comparison. Wesnes et al. [11] directly compared participants from 4 medical schools whilst Tolnai et al. [20] and Pearson et al. [18] used only 2 medical schools to form their study population. It is important to note that using graduates from multiple centres potentially increases the number of confounding variables in career preference. Of the remaining six studies, five studies directly compared graduates from PBL vs. non-PBL courses from the same medical school. Matsui et al. [9] examined different graduating classes from Tokyo Women’s Medical University. They compared graduates enrolled in the traditional curriculum versus the PBL curriculum, which was started in 1990.

There are multiple potential confounding variables when investigating factors influencing career choice. Only one of the eleven included studies (Wesnes et al. [11]) used a statistical method to attempt to adjust for confounding variables. Three further studies used matching of participants in an attempt to limit confounding. Of the remaining studies, there is no specific mention of the use of any technique to limit confounding.

### Study findings

A summary of the results is presented in Table 2. Overall there were seven out of eleven studies that showed no significant differences in career choice between graduates from a PBL vs. non-PBL curriculum. Most notably, Ford et al. [4] conducted a large study in Canada with 14,370 graduates, and this research group concluded that there were no significant differences specifically for choosing a pathology residency programme between graduates from medical schools with a predominantly PBL curriculum in comparison to those with a non-PBL curriculum. Mennin et al. [17] compared two curricula with 120 graduates in the USA. One of their tracts was geared towards Primary Care (PBL based curriculum), and the other was a traditional curriculum comprising of lectures and experiments. They found that although 12% more graduates from the Primary Care tract chose a Primary Care speciality, this was not significant (P = 0.43). A similar early study by Moore et al. [15] looked at 297 graduates from Harvard medical school comparing their New Pathway (PBL) tract to their Traditional (Non-PBL) tract. They found that there were 13% more PBL graduates choosing a speciality in Primary Care although not significant (P ≥ 0.05). Wesnes et al. [11] conducted a study looking at differences between graduates from the medical schools in Norway. This was a moderately sized study looking at 1770 graduates. They found that speciality choice was not significantly associated with the type of curriculum undertaken at medical school. Woodward et al. [19] conducted one of the first studies looking at this association in 1987. This study looked at 2028 graduates and compared McMaster’s PBL based curriculum with other Canadian non-PBL medical schools. It was found that there were no significant differences in speciality choice between the two curricula. They did, however, find that PBL graduates held a greater number of certificates in family medicine. Matsui et al. [9] conducted a small study of 468 participants in Japan comparing graduates from two tracts (PBL & Non-PBL curriculum) looking at differences in speciality choice. Although this study found that more non-PBL graduates were working in primary care (9%) there was no significance mentioned and no associated P values.

**Table 2:** Summary of study results including the outcome of each study in relation to the aims of this review

Reference (year)	Outcome measures	Results summarised	Does PBL make a student more likely to specialise in a particular career?	P value
Ford et al. [4]	Pathology residency program	No significant difference between PBL/non-PBL groups for choosing a career in pathology	No	–
Kaufman et al. [14]	Future specialty choice	PBL students who were interested in family medicine at medical school retained this interest at graduation more than Non-PBL students (42 vs 29%). By graduation 39% of PBL students switched career preference to primary care vs 14% of non-PBL students. Retention of interest in other specialties showed no significant difference between programs	Yes	Maintained family practice as choice: 0.05 switched to family practice: 0.05
Matsui et al. [9]	Specialty choice	23.7% of physicians who underwent a PBL curriculum (PBL +) were working in primary care or community care vs 31.4% of non-PBL (PBL −) physicians. 61.6% of PBL + physicians were working in ‘specialist fields’ vs 61.4% of PBL − physicians. 14.7% of PBL + physicians were working in ‘other disciplines’^a^ vs 7.1% of PBL − physicians	No	–
Mennin et al. [17]	Family practice specialty	79% of physicians from a PBL curriculum were working in primary care or a mixture of primary care/non-primary care vs 67% from a non-PBL curriculum	No	0.43
Moore et al. [15]	Specialty choice	58% of physicians from PBL curriculum worked in primary care vs 45% of Non-PBL physicians although this is not a significant difference	No	>0.05
Moore-west et al. [16]	Residency choice	Significantly increased numbers of graduates from PBL curriculum chose a primary care residency	Yes	0.025
Pearson et al. [18]	Specialty choice	57.1% of physicians from a PBL curriculum were working in primary care or psychiatry vs 44.7% of non-PBL physicians	Yes	0.0001
Peters et al. [10]	Specialty choice	40% of physicians from a PBL curriculum were working in primary care or psychiatry vs 18% of non-PBL physicians	Yes	<0.05
Tolnai et al. [20]	Family practice specialty	45.5% of physicians from a PBL curriculum were working in primary care vs 56.4% of physicians from a non-PBL curriculum	No	0.05
Wesnes et al. [11]	General practice specialty	Curriculum type (PBL vs Non-PBL) was not significantly associated with a primary care career choice	No	>0.05
Woodward et al. [19]	Specialty choice	Primary care career choice similar in PBL vs non-PBL curriculum. Graduates from PBL curriculum went on to hold a greater number certificates in family medicine	No	–

^a^PBL schools were defined as such following interviews with representatives from each of Canada’s 13 english-language medical schools

Tolnai et al. [20] compared 342 graduates from two Canadian medical schools, McMaster University (PBL) and University of Ottawa (Non-PBL). They found that there were significantly fewer graduates from a PBL curriculum working in primary care (P = 0.05). This was the only study showing this association. The authors suggest that although there is a number of influencing factors on graduating doctors’ career choice, that the actual medical school admission should be considered as a factor. Schools with non-traditional PBL-based learning may recruit students with different interests and inclinations.

Despite the majority of studies finding no correlation between PBL and a career in primary care, the smaller group of studies that did find a correlation included Peters et al. [10]. This group conducted a randomised controlled trial where 100 students were randomly assigned to either a ‘new pathway’ (PBL) curriculum or ‘traditional’ (Non-PBL) curriculum. It was found that 22% more PBL graduates were working in either primary care or psychiatry than Non-PBL graduates. This was found to be statistically significant (P ≤ 0.05). Another study also found a correlation between PBL and working in general practice. Pearson et al. [18] conducted a large study comparing 2469 students from two Australian medical schools with different curricula. It was found that significantly more graduates from the PBL curricula (Sydney) worked in primary care or psychiatry at follow-up (P = 0.0001). Lastly, Moore-West et al. [16] conducted a small study with a population of 38 subjects based at the University of New Mexico comparing their PBL and Non-PBL tracts. The study found that significantly more PBL graduates chose a primary care residency than Non-PBL graduates (P = 0.025).

Finally, there was one study that examined current medical students. Kaufman et al. [14] looked at self-reported student interest in specific specialities and how this changes throughout medical school with a PBL/Non-PBL curriculum. They found that significantly more PBL students retained an interest in a career in primary care (P = 0.05) and significantly more PBL students switched to an interest in primary care during medical school (P = 0.05).

## Discussion

Overall, the evidence to date specifically looking for a correlation between a PBL curriculum and speciality type is limited. Seven out of the 11 studies found no significant differences in speciality choice between different curricula. One study did not state the significance of its results. Only one study found that a Non-PBL curriculum is associated with an increased number of graduates pursuing a career in primary care. Three studies showed an increased number of PBL graduates pursuing a primary care or psychiatry residency choice.

### Why would PBL predispose to specific career paths?

It could be argued that problem-based learning may appeal to students with different personality characteristics due to its open and group-based nature. Understandably this learning style isn’t for everyone and Holen et al. [21] found that students that were outgoing, curious, sociable and conscientious appreciated a PBL curriculum. In turn, different career paths may appeal to graduates with different personality traits. Bexelius et al. [22] found that graduates in Sweden choosing a surgical career path scored higher in conscientiousness than those choosing other specialities. It has been hypothesised that problem-based curricula may be more likely to produce graduates who select primary care careers [23]. Furthermore, ‘Primary Care/Community tracts’ have been implemented at multiple universities with many using a PBL based approach. Regardless of the above, the simple existence of two contrasting types of medical curriculum deserves to be compared and contrasted with an important outcome such as speciality choice.

### Limitations of included studies

The evidence identified within this review is varied in geographical location, population size and outcome measures. Population size was <500 in six of the studies and in four larger studies there was a population size of >1000. Small sample sizes act to limit the power of these studies, affecting the external validity of the study results. This is highlighted when comparing Moore et al. [15] and Pearson et al. [18]. These studies were of different designs (RCT vs Cohort) and different sample sizes (297 vs 2469). This meant that although the percentage differences between groups were very similar, Moore et al. [15] found no significant difference whilst Pearson et al. [18] did find a significant difference.

Subjects for 6/11 studies were taken from only one medical school. This increases the risk of selection bias and ideally, we would have included multicentre studies. Outcome measures of the studies varied. Although all studies reported on current or future speciality choice of the graduates involved, some studies looked at this broadly whilst others focused on specific specialities such as Pathology or Family Medicine.

### Limitations of the review

Due to the limited body of relevant evidence, we could only include 11 studies, making this a relatively small review. Additionally, we had multiple studies from the same medical school with three out of 11 studies being based in University of New Mexico, two at McMaster University and two at Harvard Medical School. There are inherent difficulties in attributing a curricula type to speciality choice. PBL as an idea can be used in various different ways and to a lesser or greater extent within a curriculum. There are also a large number of confounding factors influencing speciality preference that were not adjusted for in all studies.

### Other reviews

This is the only literature review with a single focus of evaluating the correlation between curricula type and speciality choice. A previous review conducted by Albanese et al. [24] in 1993 on PBL outcomes found similar results but also had the problem of a limited evidence base.

### Conclusions and applicability

With the limited evidence available at present, this review can conclude that there is no significant association between curriculum type and career choice. Overall, 7/11 studies did not show a correlation between PBL and a specific speciality choice such as family medicine. Proponents of PBL as a way to encourage doctors to pursue a career in under-staffed specialities such as family medicine will note that there was an increased association between a PBL curriculum and family medicine in three studies within this review. Of these studies however, it is important to emphasise that one was a randomised controlled trial and one was a relatively large observational trial. The ideal way to clarify these conflicted results would be through a multi-centre, multi-country randomised-control trial with a large study population. Although PBL is used frequently worldwide, it is still a relatively new concept in many parts of the world and studies on PBL have grown substantially since its inception. Unfortunately, our knowledge on its association with speciality choice is still limited and evidence has only been conducted in a small selection of the countries that use PBL in their medical education. The findings of this study aim to clarify current knowledge on the subject and may be particularly informative to countries such as the UK or the US where there is a shortage of general physicians. New longitudinal studies looking at this association with good power and that adequately adjust for confounding variables would be welcomed.

## Abbreviations

PBL: problem-based learning
CaRMS: Canadian resident matching service
RCT: randomised controlled trial
